# Characteristics of Pre-Lens Tear Film Behavior in Eyes Wearing Delefilcon A Silicone Hydrogel Water Gradient Contact Lenses

**DOI:** 10.3390/diagnostics13243642

**Published:** 2023-12-12

**Authors:** Norihiko Yokoi, Yuki Furusawa, Hiroaki Kato, Natsuki Kusada, Chie Sotozono, Petar Eftimov, Georgi As. Georgiev

**Affiliations:** 1 Department of Ophthalmology, Kyoto Prefectural University of Medicine, Kyoto 602-8566, Japan; 2 Department of Cell and Developmental Biology, Faculty of Biology, St. Kliment Ohridski University of Sofia, 1164 Sofia, Bulgaria; peftimov@uni-sofia.bg; 3 Department of Optics and Spectroscopy, Faculty of Physics, St. Kliment Ohridski University of Sofia, 1164 Sofia, Bulgaria; ggeorg@phys.uni-sofia.bg

**Keywords:** contact lens discomfort, silicone hydrogel water gradient contact lens, pre-lens tear film, pre-lens tear meniscus, stability of tear film, contact lens wettability, material property

## Abstract

The pre-lens tear film (PLTF) over (i) delefilcon A silicone hydrogel water gradient (WG; 33–80% from core to surface) contact lenses (CLs) (SHWG-CLs) and (ii) subjects’ own non-WG soft CLs (SCLs) (SO-SCLs) was studied in 30 eyes of 30 subjects to assess the hypothesized PLTF stabilization over SHWG-CLs. In both eyes, delefilcon A SHWG-CLs (DAILIES TOTAL1^®^; Alcon, Fort Worth, TX, USA) or SO-SCLs were worn. After 15 min of wearing each lens, the tear meniscus radius (TMR, mm), lipid-layer interference grade (IG) and spread grade (SG), and non-invasive breakup time (NIBUT, seconds) were evaluated and compared between the SHWG-CLs and the SO-SCLs. The comparison between the SHWG-CL and SO-SCL groups (SHWG-CL and SO-SCL, mean ± SD) revealed that TMRs temporarily decreased and reached a plateau value after 15 min (0.21 ± 0.06; 0.21 ± 0.06) compared to the value prior to CL insertion (0.24 ± 0.08; 0.25 ± 0.08), with no significant difference between the two groups. The NIBUT, IG, and SG values after 15 min of wearing the CLs were (9.7 ± 3.7; 4.7 ± 4.2), (1.0 ± 0.2; 1.8 ± 1.0), and (1.1 ± 0.4; 1.9 ± 1.5), respectively, and all values were significantly better in the SHWG-CL group (*p* < 0.0001, *p* = 0.0039, and *p* < 0.0001, respectively). We found that compared to the SO-SCLs, the maintenance of the PLTF on the SHWG-CLs was supported by the thicker and more stable PLTF.

## 1. Introduction

It is reported that nearly 50% of the more than 140 million contact lens (CL) wearers worldwide experience discomfort when wearing their lenses [1,2], and according to the findings in a study by the Tear Film & Ocular Surface Society (TFOS) [3], CL discomfort (CLD) is defined as episodic or persistent adverse ocular sensations of varied severity related to soft CL (SCL) wear, with or without visual disturbance, resulting from reduced compatibility between the CL and the ocular environment, which can ultimately lead to decreased wearing time and discontinuation of SCL wear. Moreover, CLD is associated with (i) factors related to the SCL itself, including material properties, design, fitting/wearing condition, and care of the lens; (ii) internal factors such as age, gender, and ocular and/or general diseases of the wearers; and/or (iii) external factors such as tear film (TF) stability, blinking condition, outer environment, humidity, and air condition.

When an SCL is worn, the tear menisci are divided into the (i) pre-lens and (ii) post-lens tear menisci (TM) (PLTM and PoLTM, respectively), and tear fluid in the PLTM is used for pre-lens TF (PLTF) formation [4,5]. The thickness of the TF aqueous layer is reportedly proportional to the radius of the lower TM [6,7]. Thus, when SCLs are worn, the aqueous layer thickness of the PLTF becomes less than that of the pre-corneal TF (PCTF) [4]. Accordingly, the PLTF often shows TF breakup (BU) similar to that observed in patients afflicted with aqueous-tear-deficient dry eye (DE), in which TF BU occurs within the lower region of the cornea [4,8,9,10,11]. Moreover, the wettability of the SCL surface when measured by water contact angle (CA) is generally less than that of the corneal surface [12,13]. Therefore, the aqueous layer deposited on the SCL surface at the time when the eye is opened is expected to be thinner and less stable than that over the corneal surface, as manifested by the higher (i.e., compared to the PCTF) PLTF thinning rate [14], which also facilitates the BU of the PLTF. In addition, once it happens, the BU of the PLTF is expected to expand more rapidly due to the lower wettability of SCLs than that of the cornea [4,12,13,15]. These material-related SCL properties are responsible for PLTF instability, which is a conclusive key point in the findings of the TFOS pathophysiology report [3,16].

We previously reported [4] that within 15 min of SCL wear, the simultaneous decrease in the PLTM radius and PLTF thickness leads to diminished PLTF stability, which after several hours of SCL wear can result in CLD via the mechanism of increased friction between the eyelid wiper [17] and the SCL surface, as manifested by lid-wiper epitheliopathy [17,18] and bulbar conjunctival epithelial damage [4,19]. In this mechanism, other factors such as ocular surface (OS) inflammation related to friction and the design and/or fitting of the SCL might be involved in CLD [3].

Currently, the use of silicone hydrogel (SH) CLs (SHCLs) is increasing, and compared to CLs constructed with conventional hydrogels, SHCLs are somewhat hydrophobic and can promote PLTF instability [20,21]. Thus, an increase in SCL wettability and/or lubricity between the SCL and lid-wiper region is recommended to prevent an increase in CLD [22,23]. Indeed, recent advancements in the modification of the surface of SHCLs has improved the wettability and lubricity of the lenses via the development of a layered water gradient (WG);, i.e., the thus-named silicone hydrogel WG CLs (SHWG-CLs). As our recent in vitro experiments on the continuous blink-like exposure of SHWG-CLs to dryness mimicking the in vivo condition have demonstrated [22,23], the delefilcon A SHCL (DAILIES TOTAL1^®^, Alcon Laboratories, Inc., Fort Worth, TX, USA) was found to have superior wettability and lubricity for 16 h (as evaluated by the CA and friction coefficient, respectively) compared to non-WG SHCLs, including narafilcon A, senofilcon A, and stenfilcon A SHCLs. Delefilcon A has a characteristic WG structure, i.e., SH core (water content: 33%) coated in a 6 µm thick hydrophilic polymer (water content: 80%), and this hydrophilic polymer modification of the SH surface may be one factor contributing to the superior performance of the delefilcon A SHCL. However, although the enhanced wettability and lubricity of the delefilcon A SHCL has been demonstrated in vitro, it remains to be seen as to (1) whether and how the material properties of a SHWG-CL will influence PLTF dynamics (i.e., (i) the structure and spread of the TF lipid layer (TFLL), and (ii) the PLTF stability, i.e., BU time (BUT) and the subsequent post-BU disturbance of the PLTF structure) and (2) whether PLTF performance over the delefilcon A SHCL will be superior compared to that over the non-WG SHCL. Therefore, in this study, the in vivo effects of delefilcon A on tears and the PLTF was compared to the effects exerted on tears and the PLTF by each subject’s own SCL (SO-SCL) that did not utilize WG technology. Moreover, PLTF dynamics and stability were evaluated in terms of (1) PLTF structure and TF TFLL spread and non-invasive BUT (NIBUT, seconds) of PLTF assessed via the use of a video-interferometer (VI) [24,25], and (2) a newly developed video-keratograph (VK) parameters, the Meyer’s ring reflection disturbance value (DV), and the increase rate of DV (IRDV), which report not only on PLTF BU but also on the spatiotemporal expansion and distribution of BU regions across the SCL surface once BU occurs [26,27].

## 2. Materials and Methods

The protocols of this comparative cross-sectional study were approved by the Institutional Review Board of Kyoto Prefectural University of Medicine, Kyoto, Japan (Approval No. ERB-C-1920-2). The study was conducted in accordance with the tenets set forth in the Declaration of Helsinki, and written informed consent was obtained from all subjects prior to their involvement in the study.

### 2.1. Subjects

This study involved 30 eyes (28 right eyes and 2 left eyes) of 30 Japanese regular SCL wearers (12 males and 18 females; mean age: 33.3 ± 9.8 (mean ± SD) years) who resided in the city of Kyoto. Prior to enrollment in the study, all subjects confirmed no SCL wear on the day of initial examination before the start of the study and no eye drop use for at least 1 h prior to the initial examination. Subjects excluded from the study were those with DE that requires treatment when the SCL is not worn; those diagnosed with lid margin disease, including marginal blepharitis and meibomian gland dysfunction, based on the Japanese diagnostic criteria [28]; those diagnosed with an eyelid disease such as blepharoptosis, lagophthalmos, blepharospasm, entropion, or ectropion; and those with severe conjunctivochalasis or any history of eye surgery, including for the puncta, OS diseases, the eyelid, glaucoma, keratoconus, pterygium, filamentary keratitis, and lid-wiper epitheliopathy. Moreover, all subjects deemed ineligible for involvement in this study based on the above-described reasons, or other reasons, were excluded via consensus by three ophthalmologists (N.Y., H.K., and N.K.) following a review of the data. The SO-SCL usually used by the subjects were 14 SHCLs (excluding the delefilcon A SHCL) made from SH materials and 16 SCLs made from hydrogel materials (Table 1).

### 2.2. Clinical Assessment

#### 2.2.1. Assessment of Tear Volume by Video-Meniscometry

The TM radius (TMR, mm) was measured at the central lower lid margin with a video-meniscometer (VM) equipped with an illuminated target with horizontal stripes. The line width in the image of the target reflected at the TM was used to calculate the TMR using the concave mirror formula [28,29,30]. Reportedly, the TMR is indicative not only of tear volume at the TM but also of the total tear volume over the OS, and it is theoretically also associated with the aqueous layer thickness of the PCTF [6,7]. Indeed, it is clinically shown that a significant correlation exists in vivo between TMR and total tear volume [30], tear film thickness [6,31,32], and stability [4,33,34,35]. Thus, TMR is implemented as an overall indicator of PLTF quality.

When an SCL is worn, it is expected that the tear volume will be transiently increased via the transport of excess water from the blister pack, which decreases with time, thus reaching a plateau value representing the baseline. In our previous in vivo studies, TMR was found to reach the baseline after 5 min of SCL wear [4,36]. Thus, in this in vivo experiment, TMR was measured before and after 5, 10, and 15 min of SCL wear to determine the baseline as the most appropriate time for assessing PLTF behavior. In this study, TMR was measured 3 times, and then, averaged, with the final value then used for the analysis.

#### 2.2.2. Assessment of PCTF and PLTF Dynamics and Stability by Video-Interferometry

Using a VI (DR-1^®^; Kowa Co., Ltd., Nagoya, Japan), the interference grade (IG: Grades 1, 2, 3, 4, and 5) [4,36,37] and spread grade (SG: Grades 1, 2, 3, 4, and 5) [8] were evaluated with reference to the grading system in which the observed area corresponded to a 2.3 mm (vertical) × 3.2 mm (horizontal) rectangular area (high magnification mode of the VI) for the IG and a 6.8 mm (vertical) × 8.8 mm (horizontal) rectangular area (low magnification mode of the VI) for the SG. IG reflects the thickness of the TF aqueous layer and suggests the severity of aqueous tear deficiency, in which greater grades correspond not only to a greater severity of aqueous tear deficiency but also to a thinner TF aqueous layer, with Grade 5 indicating the most severe aqueous deficiency or a lack of aqueous TF [4,36,37]. During SCL wear, the PLTF becomes thinner than the PCTF due to the division of the original TM when the lens is inserted [4,5,36], which results in a smaller TMR, thus suggesting a thinner PLTF [6,7]. As we previously reported [4,36,37], in Grade 3 of the PLTF IG, light interference from the thinner aqueous layer can be observed together with that from the TFLL, and in Grade 2 of the IG for the PLTF and PCTF, light interference can be obtained only from the TFLL.

In addition, SG reflects the dynamic behavior of the TFLL [8,38] and suggests the severity of aqueous tear deficiency, in which greater grades correspond not only to a greater severity of aqueous tear deficiency but also to a thinner TF aqueous layer [6,7,38], with Grade 5 indicating the most severe aqueous deficiency or a lack of aqueous TF like IG [4,36,37]. SG is graded based on the behavior of the upward spread of the TFLL (i.e., the speed and extent of that spread), being classified into 1 of the following 5 grades with a modification to our original grading system [8]: Grade 1: quick and complete (the spreading TFLL quickly reaching the upper lid margin); Grade 2: slow and partial (the spreading TFLL not reaching the upper lid margin but reaching ≥3/4 the height of the image); Grade 3: slow and partial (the spreading TFLL reaching <3/4 and ≥1/2 the height of the image); Grade 4: slow and partial (the spreading TFLL reaching <1/2 and ≥1/4 the height of the image); Grade 5: partial or no spreading of the TFLL (the spreading TFLL reaching <1/4 the height of the image). There is a significant relationship between the SGs and the TMR [8,38,39], in which greater grades reflect a lesser TMR [38,39].

Finally, the TF NIBUT was measured, and in order to avoid the effect of reflex tearing, it was measured once up until 10 s and determined to be 10 s when no BU was seen for 10 s [4,36,37,38,39,40,41].

#### 2.2.3. Assessment of PLTF Dynamics and Stability by Video-Keratography

After 15 min of SCL wear, the time-dependent change in PLTF behavior when the eye was kept open for 10 s for both the SO-SCL and delefilcon A SHCL was assessed using a VK (RET-700; Rexxam Co., Ltd., Osaka, Japan) in which custom-made software with a newly developed indicator, i.e., DV (an arbitrary unit), for assessing the blurredness of Meyer’s rings, which is a reflection of the VK Placid rings [26,27], was incorporated. For DV, total DV (TDV, sum of DV for 10 s (10 images/s)) and IRDV (DV (10 s) − DV(0 s)) while keeping the eye open for 10 s were measured.

### 2.3. Study Protocol

On the day of the initial examination, the subjects were instructed to visit our examination room with their glasses and to bring their SO-SCLs without wearing them. The eyes deemed eligible for the examinations were the eyes with CLD signs, if any; right eyes with similar CLD signs; and eyes with no CLD signs in both eyes. In the “bare” eye (i.e., the eye in which no lens was worn) that was chosen in each subject, TMR was measured and the IG, SG, and NIBUT of the PCTF were assessed.

At the subjects’ first visit, they were instructed to wear the lenses in both eyes (either the SO-SCLs or delefilcon A SHCLs with the same power and the same base curve as the SO-SCL) in a single-blind fashion, where the subjects did not know which CL was chosen for wearing, and the same examinations were performed on the eye wearing the SCL. Finally, after 15 min of wearing the SCLs, PLTF behavior was assessed with a VK.

At the subjects’ second visit, after TMR, IG, SG, and NIBUT examinations were performed on the “bare” eye, the subjects were instructed to wear different SCLs from the first visit in both eyes, and the same examinations were performed on those eyes.

In the above-described examinations, TMR was measured before and after 5, 10, and 15 min of SCL wear, while IG and SG were each measured before and after 15 min of SCL wear, and DV was assessed only once after 15 min of SCL wear, with the results of those examinations then compared between the SO-SCL and delefilcon A SHCL.

During SCL wear, each subject was questioned about subjective symptoms, if any, and the appropriateness of the fitting of the SCLs was confirmed. Moreover, after the examinations, each subject was questioned about the superiority of comfort during lens wear between the SO-SCL and delefilcon A SHCL, and after lens removal, the adverse effects on the OS were examined using a slit-lamp biomicroscope.

### 2.4. Statistical Analyses

All data in this study are shown as mean ± standard deviation (SD). The paired *t*-test was used for the comparison of TMR, NIBUT, TDV, and IRDV between the SO-SCL and delefilcon A SHCL, of the time dependent-change in TMR, and of NIBUT before and after 15 min of SCL wear. The Wilcoxon signed rank test was used for the comparison of IG and SG between the usual non-WG SO-SCL and the delefilcon A SHCL. Pearson’s correlation coefficient was used to evaluate the correlation between TDV and NIBUT. Statistical analyses were performed using JMP Pro version 15.0 statistical software (SAS Institute Inc., Cary, NC, USA) for the Microsoft Windows 10 Operating System (Microsoft Corporation, Redmond, WA, USA). A *p*-value of ≤0.05 was considered statistically significant.

## 3. Results

### 3.1. Comparison of the Time-Dependent Change in TMR

**Figure 1:**
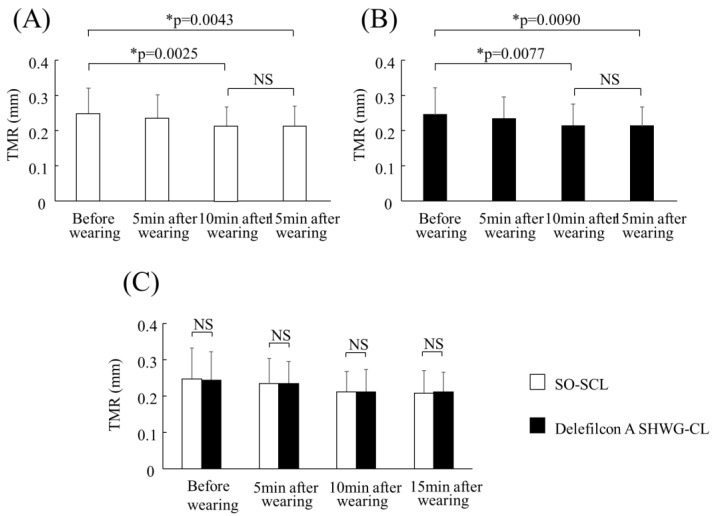
Mean tear meniscus radius (TMR) (mm) before and after 5, 10, and 15 min of wearing the subject’s own (SO) usual non-water gradient (WG) soft contact lens (SCL) (non-WG SO-SCL) (**A**) and the delefilcon A silicone hydrogel WG contact lens (SHWG-CL) (**B**). In both the SO-SCL and the delefilcon A SHWG-CL, TMR significantly decreased after 10 and 15 min of lens wear compared to that before lens wear in both SCL types (all, * *p* < 0.01), and there was no significant difference in TMR after 10 and 15 min of lens wear for both SCL types. Moreover, there was no significant difference in TMR before and after 5, 10, and 15 min of lens wear between the SO-SCL and the delefilcon A SHWG-CL (**C**).

The mean TMR (mm) of the SO-SCLs and delefilcon A SHCL eyes before and after 5, 10, and 15 min of lens wear was 0.25 ± 0.08 and 0.24 ± 0.08, 0.23 ± 0.07 and 0.23 ± 0.06, 0.21 ± 0.06 and 0.21 ± 0.06, and 0.21 ± 0.06 and 0.21 ± 0.06, respectively (Figure 1A,B). In both the usual non-WG SO-SCL and delefilcon A SHWG-CL eyes, TMR significantly decreased after 10 min (*p* = 0.0025; *p* = 0.0077) and 15 min (*p* = 0.0043; *p* = 0.009) of SCL wear compared to before SCL wear in both SCL types, and there was no significant difference in TMR after 10 and after 15 min (*p* = 0.6239; *p* = 0.7455). These findings suggest that TMR reached a plateau value after 10 min of lens wear in both lens types. As reported previously [4], at ≤5 min of SCL wear immediately after the insertion of the lens, the TMR increases by 30–80% compared to that prior to insertion due to the transport of excess water from the blister pack. However, this is a transient effect that rapidly vanishes within a few minutes of SCL wear, which is in agreement with the current data. Moreover, there was no significant difference in TMR before and after 5, 10, and 15 min of SCL wear between the SO-SCL and delefilcon A SHCL (*p* = 0.7394; *p* = 0.9511; *p* = 0.8496; *p* = 0.8151) (Figure 1C).

### 3.2. Comparison of IG, SG, and NIBUT

**Figure 2:**
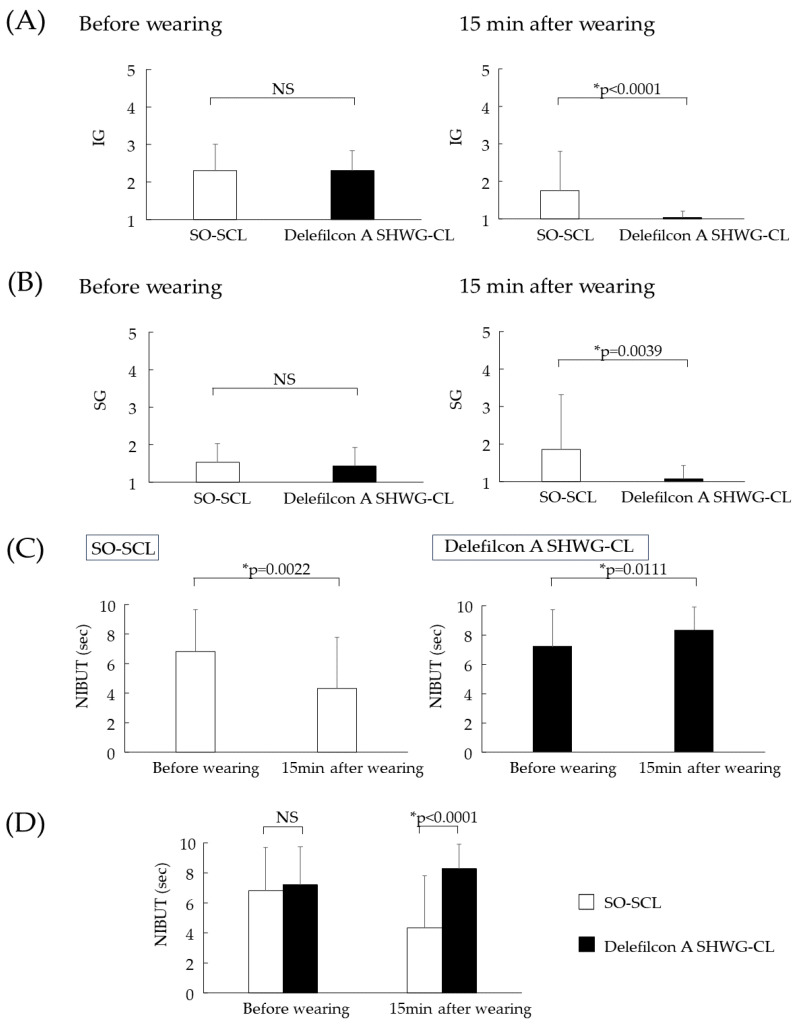
Interference grade (IG), spread grade (SG), and non-invasive breakup time (NIBUT) (seconds) for the usual non-WG SO-SCL and delefilcon A SHWG-CL before insertion of the lens and after 15 min of lens wear. Before lens insertion, there was no significant difference in IG between the “bare” (without SCL) eyes where the delefilcon A SHWG-CL or other SCL (*p* = 1.000) were subsequently fitted (**A**, Left). However, after 15 min of lens wear, significantly lower IGs were observed in the delefilcon A SHWG-CL compared with the SO-SCL (*p* < 0.0001) (**A**, Right), thus suggesting that the pre-lens tear film (PLTF) thickness over the delefilcon A SHWG-CL is significantly greater than that over the SO-SCL. Prior to lens insertion, there was no significant difference in SG between the “bare” eyes where the delefilcon A SHWG-CL or other SCL were subsequently fitted (*p* = 0.5078) (**B**, Left). However, after 15 min of lens wear, significantly lower (i.e., clinically superior) SGs were observed in the tear film lipid layer in the eyes fitted with the delefilcon A SHWG-CL (*p* = 0.0039) (**B**, Right), thus suggesting that the PLTF thickness is significantly thicker over the delefilcon A SHWG-CL than that over the usual non-WG SO-SCL. Comparison of NIBUT shows that over the usual non-WG SO-SCL, after 15 min of SCL wear, NIBUT gets shorter compared to that prior to lens insertion (**C**, Left), thus suggesting that the PLTF stability was decreased. In contrast, NIBUT over the delefilcon A SHWG-CL reveals that after 15 min of lens wear, NIBUT gets longer compared to that prior to lens insertion (**C**, Right), thus suggesting that the PLTF stability was increased. Prior to SCL wear, there was no significant difference in NIBUT between the “bare” eyes where the delefilcon A SHWG-CL or usual non-WG SO-SCL were subsequently inserted (**D**). However, after 15 min of lens wear, significantly longer NIBUT was observed over the delefilcon A SHWG-CL (* *p* < 0.0001) (**D**), thus suggesting that the PLTF stability was significantly better over the delefilcon A SHWG-CL than over the usual non-WG SO-SCL. Videos illustrating data on the dynamics and the IGs observed in the PLTFs formed on top of the WG and non-WG CLs are available in the Supplementary Files.

The numbers of cases in IG 1, 2, 3, 4, and 5 before and after 15 min, of lens wear for the SO-SCL were 2, 19, 7, 2, and 0 and 15, 11, 1, 2, and 1, respectively, while those for the delefilcon A SHWG-CL were 0, 22, 7, 1, and 0, and 29, 1, 0, 0, and 0, respectively. Thus, the mean IGs (mean ± SD) before and after 15 min of lens wear for the usual non-WGSO-SCL and the delefilcon A SHWG-CL were 2.3 ± 0.7 and 2.3 ± 0.5, and 1.8 ± 1.0 and 1.0 ± 0.2, respectively. Before SCL wear, no significant difference in IG was found between the usual SO-SCL and the delefilcon A SHWG-CL (*p* = 1.000) (Figure 2A, Left). However, after 15 min of lens wear, significantly lower (i.e., clinically superior) IG grades were found in the delefilcon A SHWG-CL eyes (*p* < 0.0001) (Figure 2A, Right), thus suggesting a significantly greater PLTF thickness in the delefilcon A SHWG-CL eyes than in the SO-SCL eyes.

The numbers of cases in SG 1, 2, 3, 4, and 5 before and after 15 min of lens wear for the SO-SCL were 14, 16, 0, 0, and 0, and 21, 1, 2, 3, and 3, respectively, while those for the delefilcon A SHWG-CL were 17, 13, 0, 0, and 0, and 29, 0, 1, 0, and 0, respectively. Thus, the mean SGs (mean ± SD) before and after 15 min of lens wear for the usual non-WG SO-SCL and the delefilcon A SHWG-CL were 1.5 ± 0.5 and 1.4 ± 0.5, and 1.9 ± 1.5 and 1.1 ± 0.4, respectively (Table 2). Prior to SCL insertion, there was no significant difference in SG between the eyes of the volunteer subjects (*p* = 0.5078) (Figure 2B, Left). However, after 15 min of lens wear, significantly lower SG grades were found in the delefilcon A SHWG-CL eyes (*p* = 0.0039) (Figure 2B, Right), thus suggesting a significantly greater PLTF thickness in the delefilcon A SHWG-CL eyes than in the SO-SCL eyes.

The mean NIBUTs for the usual non-WG SO-SCL prior to lens insertion and after 15 min of lens wear were 6.8 ± 2.8 and 4.3 ± 3.5, respectively (Figure 2C, Left), thus suggesting that the PLTF stability was decreased after 15 min of lens wear. However, the NIBUTs for the delefilcon A SHCL prior to lens insertion and after 15 min of lens wear were 7.2 ± 2.6 and 8.3 ± 2.2, respectively (Figure 2C, Right), thus suggesting that the PLTF stability was increased over the delefilcon A SHWG-CL. Prior to SCL insertion, there was no significant difference in NIBUT between the eyes where the usual non-WG SO-SCL and delefilcon A SHWG-CL were subsequently fitted (*p* = 0.5196) (Figure 2D, Left). However, after 15 min of lens wear, the delefilcon A SHWG-CL showed significantly longer NIBUT (*p* < 0.0001) (Figure 2D, Right), thus suggesting that the PLTF stability was significantly better in the delefilcon A SHWG-CL eyes than in the usual non-WG SO-SCL eyes after 15 min of lens wear. Videos illustrating the dynamics and the IGs observed in the PLTF that formed on top of the WG and non-WG CLs are available in the Supplementary Files.

### 3.3. Comparison of TDV and IRDV

**Figure 3:**
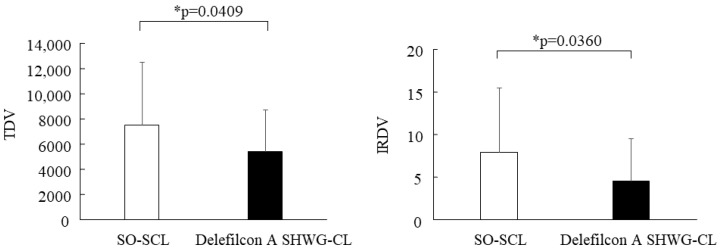
Total disturbance value (TDV) and increase rate of DV (IRDV) for the usual non-WG SO-SCL and delefilcon A SHWG-CL after 15 min of lens wear. TDV was significantly greater in the usual non-WG SO-SCL eyes than in the delefilcon A SHWG-CL eyes (**Left**). IRDV for the usual non-WG SO-SCL was significantly greater than that for the delefilcon A SHWG-CL (**Right**), thus suggesting that PLTF stability is significantly better in the delefilcon A SHWG-CL than in the usual non-WG SO-SCL.

The TDVs for the usual non-WGSO-SCL and delefilcon A SHWG-CL after 15 min of lens wear were 7521 ± 4946 and 5408 ± 3284, respectively, and TDV was significantly greater in the usual non-WG SO-SCL than that in the delefilcon A SHWG-CL (*p* = 0.0409) (Figure 3, Left). The IRDVs for the usual non-WG SO-SCL and delefilcon A SHWG-CL after 15 min of lens wear were 7.95 ± 7.56 and 4.59 ± 4.92, respectively, and IRDV was significantly greater for the usual non-WG SO-SCL than that for the delefilcon A SHWG-CL (*p* = 0.0360) (Figure 3, Right). These findings suggest that PLTF stability is significantly better over the delefilcon A SHWG-CL than over the SO-SCL.

### 3.4. Subjective Symptoms, Fitting of the SCLs, Comfort of the Eye, and OS Damage through SCL Wear

In all subjects, there were no complaints of subjective symptoms while wearing the usual non-WG SO-SCL and delefilcon A SHWG-CL, and the fitting of the delefilcon A SHWG-CL was found to be acceptable. Of the 30 subjects, after 15 min of wearing the delefilcon A SHWG-CL, 16 (53%) reported better comfort, 12 (40%) reported equal comfort, and 2 (6.7%) reported worse comfort when wearing the delefilcon A SHWG-CL in comparison to when wearing their usual non-WG SO-SCL. In the eyes of all subjects, no OS epithelial damage was observed after the removal of the lens.

## 4. Discussion

The findings in our previous in vitro study [22,23] revealed that the delefilcon A SHWG-CL has greater wettability and lubricity than other non-WG SCLs, including narafilcon A, senofilcon A, and stenfilcon A SHCLs, probably due to the material properties of the delefilcon A SHWG-CL, which is based on WG technology. Unlike the surface structure of the other SO-SCLs in this study, the SH core of the delefilcon A SHWG-CL (water content ratio: 33%) is coated in a hydrophilic polymer comprising a 6 μm thick hydrogel layer (water content ratio: 80%) [41] that effectively maintains the PLTF thickness and stability [42]. Moreover, in comparison with the other non-WG SCLs, the superior material properties that the delefilcon A SHWG-CL demonstrated in vitro [22,23] were found to have a positive effect on PLTF in vivo, which is what we expected prior to the start of the study.

In our best effort to effectively assess the effect of the material properties of the SCLs on the PLTF, while avoiding both (i) a temporal increase in tear volume due to the import of aqueous fluid from the blister pack immediately after SCL insertion and (ii) the expected time-dependent deterioration of those material properties during lens wear [22,23], we first investigated the earliest and most appropriate time to evaluate the PLTF by monitoring the time-dependent change in the PLTM radius. This was done because there is a positive relationship between the TMR and tear volume and TF thickness over the OS [6,7,30], and the findings in our previous studies demonstrated a positive relationship between the PLTM radius and PLTF stability [4,36]. In this present study, we found that after insertion of the delefilcon A SHWG-CL and the other SO-SCLs, the TMR temporarily increased for 5 min after insertion, and then, decreased and reached a baseline value within 10 min after insertion. This finding correlates well with the findings in our previous studies on hydrogel SCLs [4,36]. Accordingly, 15 min post-SCL insertion was thought to be the most appropriate and reasonable time period to evaluate the effect of the SCL’s material properties on PLTF behavior with minimal interference by the deterioration and the temporary increase in tear volume after the SCL is worn.

Moreover, the decrease in the PLTM radius post-lens insertion compared to the TMR pre-insertion was found to be identical (i.e., with no significant differences observed) between the delefilcon A SHWG-CL and the usual non-WG SO-SCL due to the fact that the original tear meniscus becomes divided when the lens is inserted [4,5]. However, after 15 min of SCL wear, the IG and SG of the TFLL were found to be of lower (i.e., clinically superior) grades over the delefilcon A SHWG-CL than over the usual non-WG SO-SCL, although the IG and SG of the PCTF were not significantly different between the eyes prior to the insertion of the lens. As has been previously reported, the enhancement of both the IG and SG also enhances the aqueous layer [4,38,43,44,45], which is probably why the aqueous stratum of the PLTF was found to be thicker over the delefilcon A SHCL than over the usual non-WG SO-SCLs. In addition, in all of the SCLs examined in this study, the TMR, which is a reservoir of tear fluid [6,7], was identical, and the increased thickness of the aqueous stratum must be explained by the superior wettability and water holding property of the delefilcon A material. This finding supports the data obtained in our in vitro study [22,23], where the delefilcon A SHWG-CL showed significantly lower water CAs, i.e., superior wettability, than the non-WG SCLs that were examined.

In this study, PLTF stability was compared between the delefilcon A SHWG-CL and the usual non-WG SO-SCLs after 15 min of lens wear in terms of (i) NIBUT assessed by VI [24,25,44] and (ii) TDV and IRDV for 10 s assessed by VK [4,36,37,40], all of which are non-invasive and objective indicators for the assessment of PCTF and PLTF stability. However, while NIBUT uses the first local TF BU as the sole indicator of PLTF instability within the VI observation area (i.e., the 6.8 mm (vertical) × 8.8 mm (horizontal) rectangle), TDV and IRDV [26,27], both of which were first used in this present study, provide additional information on PLTF instability and structure (i.e., the fraction and distribution of dry patch regions) across a wider area (i.e., the entire lens surface within the palpebral zone). Moreover, the TDV and IRDV values provide information not only on PLTF stability, but also on the wettability of the SCL surface, because lower wettability represented by lower CA may reflect upon the expansion of the BU area [46], which, in turn, results in greater TDV and IRDV. From this aspect, PLTF dynamics and stability when the eye is kept open may be more comprehensively reported by TDV and VK than by NIBUT alone, as TDV and VK are measured throughout the entire period during which the eye is open, while NIBUT is measured only until the moment when the PLTF BU occurs. In addition, in our recent review [4], we referred to the importance of SCL wettability that stops the expansion of the PLTF BU, as expansion of the BU is proportional to the third power of the SCL surface CA [46], and thus, the low water CA (around 25° when freshly removed from the blister) of delefilcon A may assure lower TDV and VK. This was indeed also suggested in previous studies, where delefilcon A maintained a two- to three-times better visual wettability grade and lower deposit grade compared to somofilcon A and narafilcon A for up to 8 h of wear [47] and increased the PLTF stability for up to 2 s while suppressing the thin aqueous layer break (i.e., an expansive BU associated with the formation of an excessively thin aqueous layer on top of the SCL) compared to narafilcon A after 5 h of SCL wear [42]. In agreement with these considerations (i.e., increased aqueous layer thickness, as manifested by superior TFLL grades, better wettability, and resistance to the expansion of dry patches of the delefilcon A surface) and with the clinical data reported by other teams [42,47,48,49], all the indicators (NIBUT, TDV, and VK) were highly correlated and showed a similar trend after 15 min of CL wear, i.e., better PLTF quality and stability over the delefilcon A SHCL compared to the usual non-WG SO-SCLs that do not have a WG two-layer structure (the typical manifestation of these trends is shown in the online Supplementary File). The fact (i) that there was no difference between the PCTF characteristics and stability in the “bare” eyes prior to the insertion of the CL, and (ii) that the TMR (a tear fluid reservoir [4,36]) was identical for all of the SCLs studied, emphasizes the importance of the SH material properties, and wettability in particular, for the clinical performance of the SCL and for the PLTF dynamics (aqueous tear deposition and thickness, TFLL structure and spread, etc.) and stability. Hence, the findings in this present study conclusively demonstrated the superiority of the surface properties of delefilcon A SHWG-CL over a variety of non-WG SCLs that the subjects usually wore, a conclusion that we expected from the findings in our in vitro studies [22,23]. Moreover, and to our surprise, the NIBUT of the PLTF after 15 min of wearing delefilcon A is longer (i.e., clinically superior) than that of the PCTF prior to the lens being inserted. Similar findings of increased stability of the PLTF as compared to the PCTF over the “bare” corneal surface were recently reported for another SHWG, lehfilcon A [50]. It is unclear as to why delefilcon A showed such superiority, as it is thought that the wettability of the healthy corneal surface should be better than that of SCLs [12,13,22,23]. Possibly, it might be that the surface of fresh WG lenses is more capable of maintaining the stability of wetting films compared to a healthy corneal surface due to the low water CA, the superior topography, and the resistance to contamination by deposits of the SHWG. However, further study is needed to prove this, as are studies of the alteration of PLTF stability compared to PCTF following extended wear of delefilcon A.

It should be noted that this study did have limitations. First, the delefilcon A SHWG-CL was only compared with the subjects’ SCL of choice, and no targeted comparison was made between delefilcon A and narafilcon A, senofilcon A, and stenfilcon A, for which comprehensive in vitro findings have been reported [22,23]. If such a comparison had been carried out, a more detailed correlation could have been obtained between the differences in the material properties and the in vivo performance of the delefilcon A SHWG-CL compared to the rest of the SCLs. Moreover, a further study should be conducted to examine how the superior PLTF aqueous layer thickness and stability of delefilcon A is maintained for a longer wearing time despite the expected deterioration of the SCL’s material properties. Supportive data in this regard include the ability of delefilcon A to maintain a superior visible graded wettability and longer NIBUT (13.4 ± 4.4 s) than filcon II-3 (11.6 ± 3.7 s; *p* < 0.001), narafilcon A (12.3 ± 3.7 s; *p* < 0.001), and somofilcon A (2.6 s shorter NIBUT than delefilcon A; *p* < 0.001) for up to 16 h of wear [47,49].

It should be noted that one limitation in this present study was the relatively small sample size of 30 individuals, as the enrollment of more volunteers was deemed unnecessary due to the preference of many CL wearers to stick to their usual CL of choice (i.e., they were reluctant to use other CL types). At the same time, *n* = 30 corresponds to the minimum number of volunteers reportedly considered sufficient for the application of the standard parametric and nonparametric statistical tests implemented in this type of study [29,33], and similar sample sizes were used in previous research on PLTF properties [48,49,50]. The good agreement of the main findings of the current study (i.e., superior PLTF dynamics and stability over the delefilcon A SHCL surface as compared to the SO-SCL and to PCTF after 30 min of SHWG-CL wear) agrees with the results reported in previous studies [42,47,48,49,50] that used diverse sample sizes (*n* = 20 to 53 subjects) comprising individuals from various ethnicities and races (i.e., Asian, Latino, and Caucasian), thus supporting the general validity of the findings in this study.

In conclusion, the findings in this study demonstrate that in comparison to the usual non-WG SO-SCLs, the PLTF aqueous layer is thicker and more stable in the delefilcon A SHWG-CL, probably due to the thicker surface hydrogel layer provided by the WG technology.

## Figures and Tables

**Table 1:** Type of non-WG lens each subject used and the corresponding number of subjects using that lens.

Groups	Characteristics	Number of Subjects
Group I	NI/LWC	1
Group II	NI/HWC	4
Group III	I/LWC	0
Group IV	I/HWC	11
Group V-A	I/NWC	0
Group V-B	NI/HWC	0
Group V-Cm	NI/LWC, ST	2
Group V-C	NI/LWC, ST/HM	1
Group V-Cr	NI/LWC, Non-ST/SIN	11

NI: non-ionic; I: ionic; NWC: no water content; LWC: low water content; HWC: high water content; ST: surface treatment; HM: hydrophilic monomer; SIN: semi-interpenetrating network.

**Table 2:** The number of cases in interference grades (IGs), spread grades (SGs) 1, 2, 3, 4, and 5, and the mean IG and SG (mean ± SD) before and after 15 min of lens wear for the SO-SCL and the delefilcon A SHWG-CL.

	SO-SCL		Delefilcon A SHWG-CL	
Grade	before Lens Wear	15 min after Lens Wear	before Lens Wear	15 min after Lens Wear
*IG*				
1	2	15	0	29
2	19	11	22	1
3	7	1	7	0
4	2	2	1	0
5	0	1	0	0
mean ± SD	2.3 ± 0.7	1.8 ± 1.0	2.3 ± 0.5	1.0 ± 0.2
*SG*				
1	14	21	17	29
2	16	1	13	0
3	0	2	0	1
4	0	3	0	0
5	0	3	0	0
mean ± SD	1.5 ± 0.5	1.9 ± 1.5	1.4 ± 0.5	1.1 ± 0.4

## Data Availability

The data that support the findings in this study are available on reasonable request from the corresponding author.

## Supplementary Materials

The following supporting information on IG, SG and NIBUT in Figure 2, and TDV and IRDV in Figure 3 can be downloaded at: https://www.mdpi.com/article/10.3390/diagnostics13243642/s1. The Supplementary material to Figure 2 compares the distinct impact of the subject’s own usual non-water gradient soft contact lens (non-WG SO-SCL; lotrafilcon A (Group: V-Cm)) and SHWG-CL (delefilcon A) on the tear volume, precorneal tear film and corneal surface parameters of a representative case (a 28-year-old female, right eye) before and 15 min after 15 SCL wear: tear meniscus radius (TMR) (Figure S1); interference grade (IG) (Figure S2); spread grade (SG) and non-invasive breakup time (NIBUT) (Figure S3); disturbance value (DV) change after the eye is kept open for 10 s (Figure S4); total disturbance value (TDV) and increase rate of disturbance value (IRDV) (Figure S5) after the eye is kept open for 10 s. Note that after 15 min of SCL wear in the same eye (right eye) of the same subject on different days, the PLTF is thinner (IG: Grade 2) and unstable (SG: Grade 2; NIBUT: 0 s) in the usual non-WG SO-SCL compared to that (IG: Grade 1; SG: Grade 1; NIBUT: 10 s) in the delefilcon A SHWG-CL despite the similarity of the meniscus tear volume between the two lenses due to the similarity of the TMR values. Video S1: A video-recorded interference image of the representative case in Figure 2 (left) of the Supplementary Materials reflecting the pre-lens tear film (TF) (PLTF) thickness. Light interference from the aqueous layer of the PLTF can be observed together with that from the tear film lipid layer (TFLL), thus indicating that the PLTF is relatively thinner, and the interference grade (IG) corresponds to Grade 2. Video S2: A video-recorded interference image of the representative case in Figure 2 (right) of the Supplementary Materials reflecting the PLTF thickness. Light interference can be observed only from the TFLL, thus indicating that the PLTF is normal in thickness, and the IG corresponds to Grade 1. Video S3: A video-recorded interference image of the representative case in Figure 3 left of the Supplementary Materials reflecting the PLTF dynamics and stability. The TFLL spread is slow and partial (i.e., the spreading TFLL not reaches the upper lid margin and only reaches up to ≥3/4 the height of the image), and immediately after the eye is opened, the PLTF breakup can be observed (especially in the upper-most area of the image), corresponding to a non-invasive breakup (BU) time (NIBUT) of 0 s. Video S4: A video-recorded interference image of the representative case in Figure 3 (right) of the Supplementary Materials reflecting the PLTF dynamics and stability. The TFLL spread is quick and complete (i.e., the spreading TFLL quickly reaches the upper lid margin), and no TF BU can be observed at 10 s after the eye is kept open, corresponding to an NIBUT of 10 s.

## Abbreviations

BU: breakup; CA: contact angle; CL: contact lens; CLD: contact lens discomfort; DE: dry eye; DV: disturbance value; IG: interference grade; IRDV: increase rate of disturbance value; NIBUT: non-invasive breakup time; OS: ocular surface; PCTF: pre-corneal tear film; PLTF: pre-lens tear film; PLTM: pre-lens tear meniscus; SCL: soft contact lens; SG: spread grade; SH: silicone hydrogel; SHCL: silicone hydrogel contact lens; SHWG-CL: silicone hydrogel water gradient contact lens; TDV: total disturbance value; TF: tear film; TFLL: tear film lipid layer; TFOS: Tear Film & Ocular Surface Society; TM: tear meniscus; TMR: tear meniscus radius; VI: video-interferometer; VK: video-keratograph; VM: video-meniscometer; WG: water gradient.
