# Supplementary material for: Characteristics of Pre-Lens Tear Film Behavior in Eyes Wearing Delefilcon A Silicone Hydrogel Water Gradient Contact Lenses
Source: Diagnostics (Basel). 2023 Dec 12;13(24):3642. doi: 10.3390/diagnostics13243642 (PMC10742534; doi:10.3390/diagnostics13243642)

Figure S1. Comparison of TMR in a representative case (28 y.o. female, right eye) between usual non-WG SO-SCL and DelefilconA SHCL 15 min after each CL wear

Usual SCL

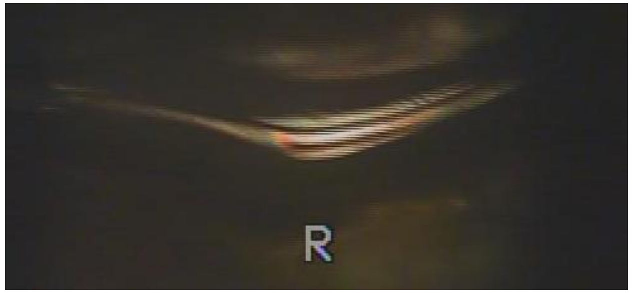

TMR=0.20445

Delefilcon A SHCL

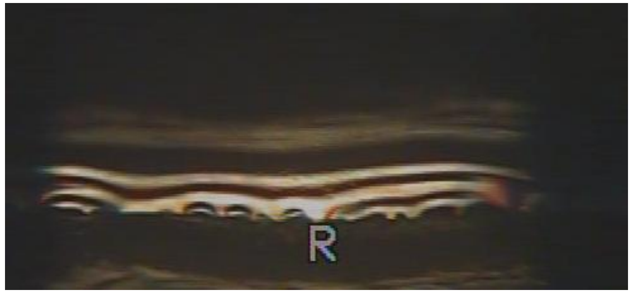

TMR= 0.19802

Figure S2. Comparison of IG in a representative case (28 y.o. female, right eye) between usual non-WG SO-SCL and DelefilconA SHCL 15 min after each CL wear

Usual SCL

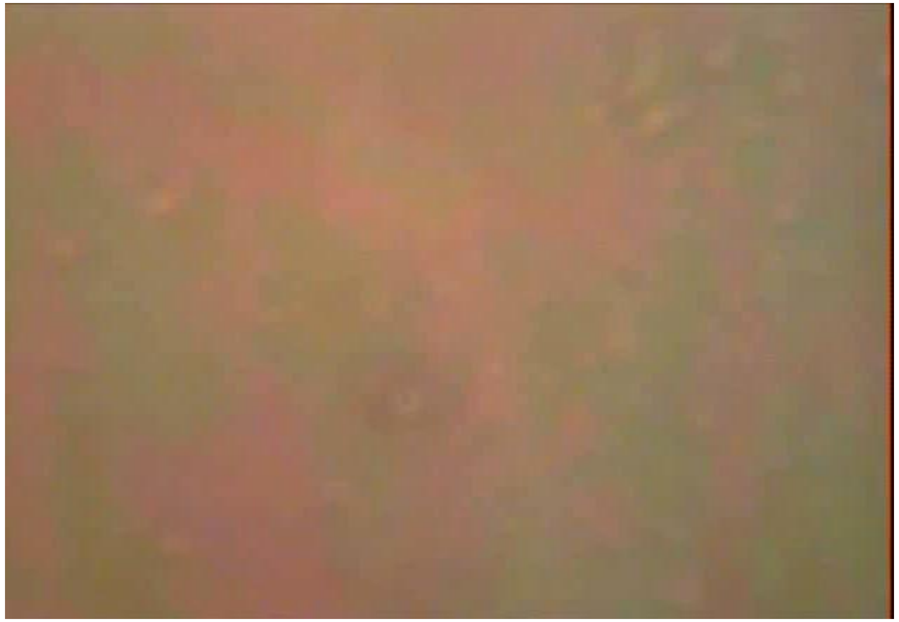

Grade 2

Delefilcon A SHCL

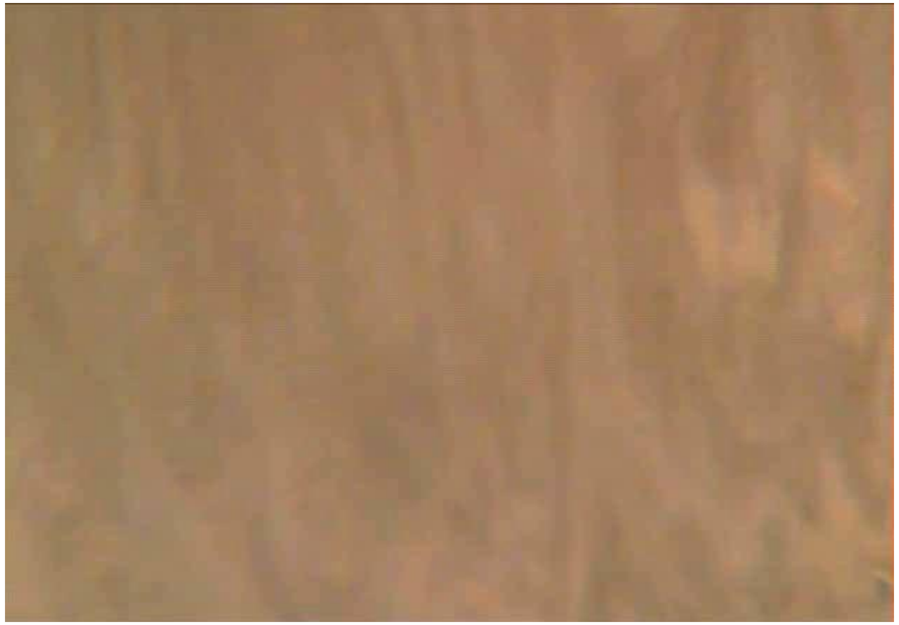

Grade 1

Figure S3. Comparison of SG and NIBUT in a representative case (28 y.o. female, right eye) between usual non-WG SO-SCL and DelefilconA SHCL 15 min after each CL wear

Usual SCL

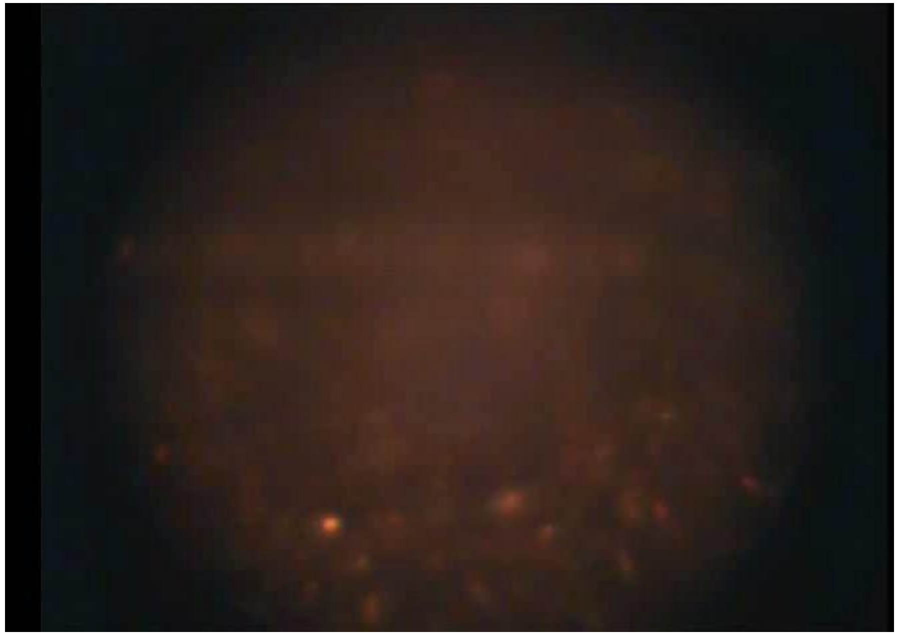

SG=2, NIBUT=0sec

Delefilcon A SHCL

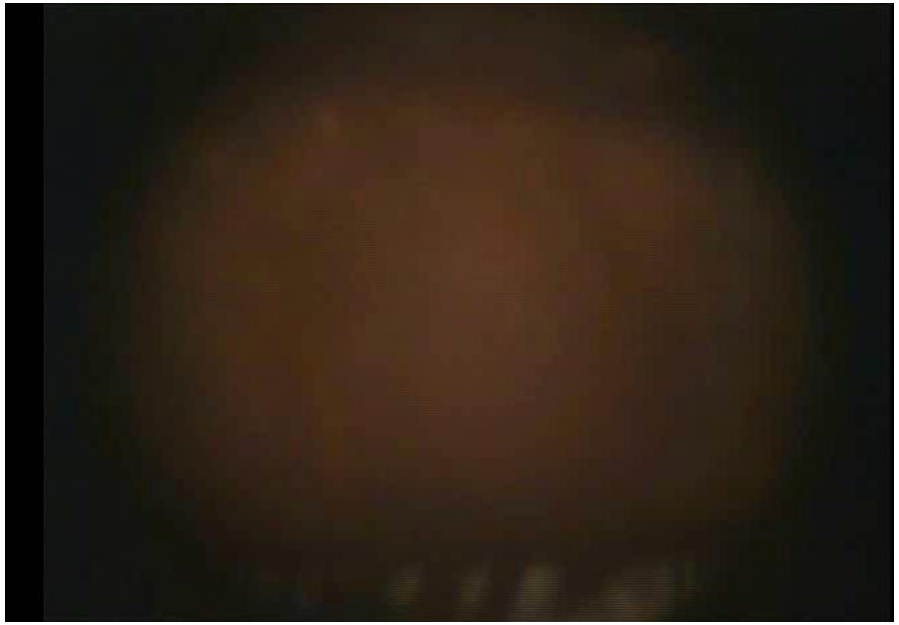

SG=1, NIBUT=10sec
(No breakup can be seen
when the eye is kept open for 10sec）

Figure S4. Comparison of DV change with time after eye is kept open in a representative case (28 y.o. female, right eye) between usual non-WG SO-SCL and DelefilconA SHCL 15 min after each CL wear

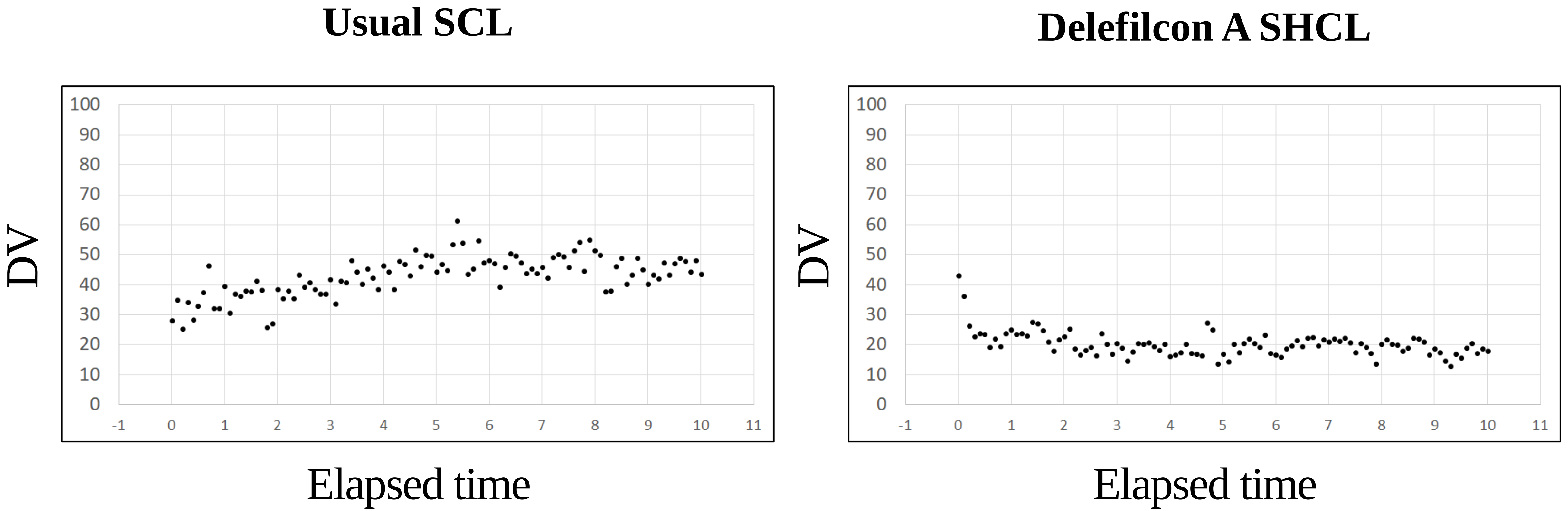

Figure S5. Comparison of TDV and IRDVin a representative case (28 y.o. female, right eye) between usual non-WG SO-SCL and DelefilconA SHCL 15 min after each CL wear

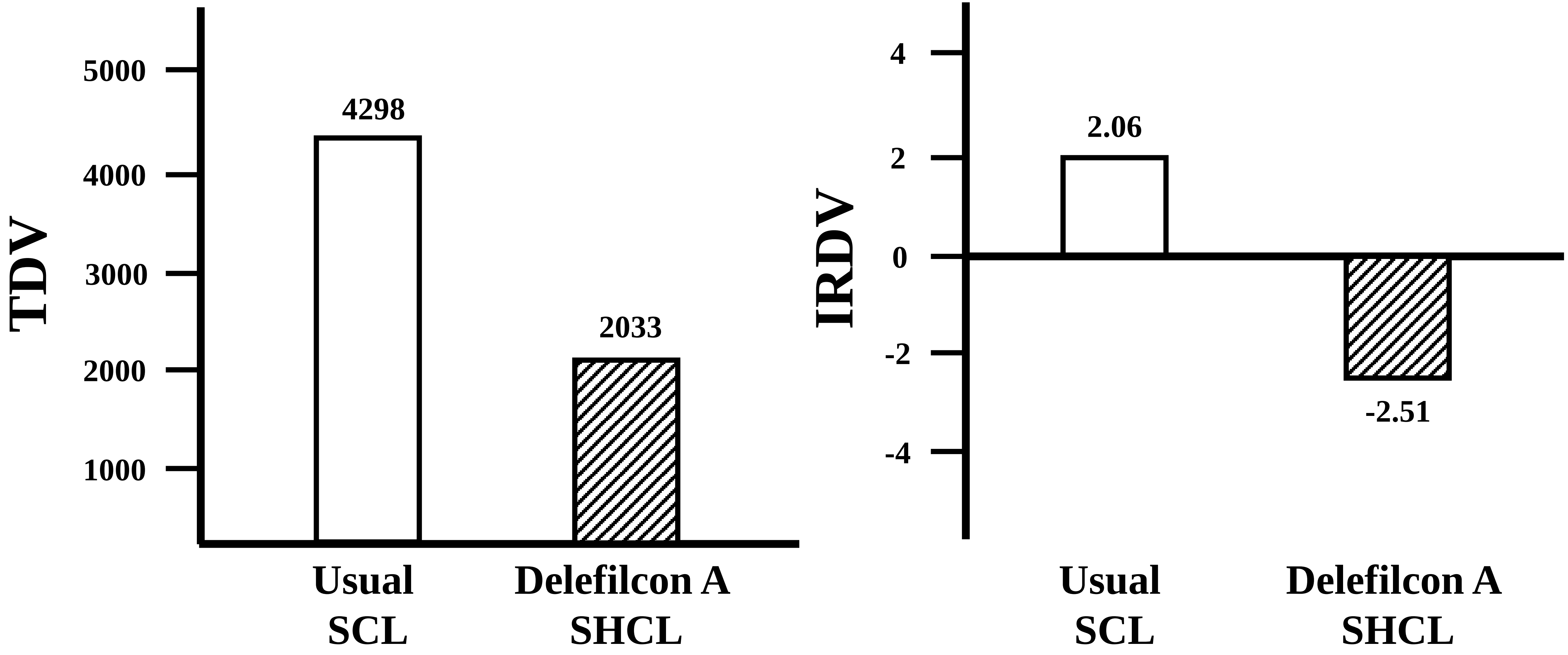

Supplement: Supplementary file 1 [file diagnostics-13-03642-s001.zip › Supplementary Materials to Figure 2.pdf]
